# PROMOTING OLDER ADULTS’ INTERGENERATIONAL COMMUNICATION AND MOTIVATION TO REGULAR EXERCISE THROUGH EXERGAMING

**DOI:** 10.1093/geroni/igz038.508

**Published:** 2019-11-08

**Authors:** Jinhui Li, Chen Li, Bing Xun Chia, Xinran Chen, Tan Phat Pham, Charles T Salmon, Yin Leng Theng

**Affiliations:** 1 Nanyang Technological University, Singapore, Singapore; 2 Ageing Research Institute for Society and Education, Singapore, Singapore

## Abstract

Exergaming has become an important part of community programs to promote regular exercise and improve well-being for healthy aging. This study examines how different types of social playing and competitive information in exergaming affect older adults’ inter-generational communication with youth, as well as their motivation to regular exercise through exergaming. A 2 (time: pre-test vs. post-test) * 3 (social play: play alone vs. play with elderly vs. play with youth) * 2 (competition: competition informed vs. non-competition informed) mixed experiment with 319 Singaporean older adults conducted over six weeks in 2018, analyzed through a series of three-way repeated ANOVAs. Results show significant three-way interaction effects among time, social type, and competitive information on older adults’ inter-generation communication (F (2, 300) = 3.206, p = .042, η2 = .021), extrinsic motivation (F (2, 301) = 3.364, p = .036, η2 = .022) and intrinsic motivation (F (2, 303) = 4.528, p = .012, η2 = .029). The inter-generational communication of participants in play with youth shows significant improvement over time in both competition conditions, while those in play with elderly was only significantly improved in competitive informed condition. Social play is found to significantly affect the changes of both intrinsic and extrinsic motivation over time, while competitive information only affects intrinsic motivation significantly. The findings make contributions to aging research and understanding of potential factors that promote inter-generational communication and adherence to regular exercise via exergaming.

